# Previous breastfeeding difficulties: an existential breastfeeding trauma with two intertwined pathways for future breastfeeding—fear and longing

**DOI:** 10.1080/17482631.2019.1588034

**Published:** 2019-03-20

**Authors:** Lina Palmér

**Affiliations:** Faculty of Caring Science, Work Life and Social Welfare, University of Borås, Borås, Sweden

**Keywords:** Breastfeeding difficulties, breastfeeding story, breastfeeding trauma, experiences, existential, fear of breastfeeding, lifeworld, hermeneutics

## Abstract

**Purpose**: Experiencing breastfeeding difficulties poses a risk for early cessation of breastfeeding and decreases the likelihood of breastfeeding a future child. To further understand breastfeeding outcomes, the aim of this study is to explain the phenomenon of breastfeeding difficulties in order to understand how women’s previous experiences of breastfeeding difficulties relate to their decisions about future breastfeeding.

**Methods**: A reflective lifeworld hermeneutical approach was adopted. The study consisted of 15 lifeworld interviews with eight women who had previously experienced difficulties with initial breastfeeding.

**Results**: Previously experienced breastfeeding difficulties represent an existential breastfeeding trauma in an individual woman’s life, from which there are two intertwined pathways for future breastfeeding: a fear of breastfeeding, which renders the idea of future breastfeeding unthinkable, and a longing for breastfeeding, which increases the likelihood of future breastfeeding. Fear and longing are intertwined in ambiguous ways in an individual woman’s life.

**Conclusion**: Women with previous breastfeeding difficulties may bring negative breastfeeding experiences with them, which are etched into the woman’s being as a mother as an embodied memory. A lifeworld-led caring science perspective as a foundation for care can contribute to the development of caring practices, which grasp the existential nature of the breastfeeding trauma.

## Introduction

Almost all new mothers in Sweden (95%) initiate breastfeeding, but statistics from Sweden show that only 76% exclusively breastfeed for 1 week after birth, and only 63% exclusively breastfeed for 2 months after birth (National Board of Health and Welfare, 2018). Previous research has shown that nearly 30% of new mothers in Sweden experience difficulties, such as sore nipples, mastitis and suckling problems among others, when initiating breastfeeding, (Almgren-Tangen, Bergman, Dahlgren, Roswall, & Alm, 2012), and that this is a major cause of early breastfeeding cessation (Almgren-Tangen et al., 2012; DiGirolamo, Thompson, Martorell, Fein, & Grummer-Strawn, 2005; Odom, Li, Scanlon, Perrine, & Grummer-Strawn, 2013). International breastfeeding statistics report an incidence of breastfeeding difficulty for 27–87% of women, (Chaput, Nettel-Aguirre, Musto, Adair, & Tough, 2016), making such difficulties a problem for women worldwide. Previous negative breastfeeding experiences and difficulties have a strong influence on breastfeeding initiation and duration for subsequent children and are risk factors for not breastfeeding a future child at all. For example, a multiparous woman who breastfed her first child for a short period of time tends to do the same with her next child or does not attempt breastfeeding at all (Bai, Fong, & Tarrant, 2015; Nagy, Orvos, Pál, Kovács, & Loveland, 2001; Phillips, Brett, & Mendola, 2011; Schafer, Campo, Colaizy, Mulder, & Ashida, 2017). Mothers’ intentions to never initiate or to stop breastfeeding early were found to be intentions that were fulfilled (DiGirolamo et al., 2005). An initial negative breastfeeding experience was also identified as a significant and measurable risk factor for early termination, a finding that is important for healthcare professionals to consider when developing strategies for care for breastfeeding mothers. Larsen and Kronborg (2013) found that mothers who had breastfeeding difficulties, which forced them to stop breastfeeding earlier than they had planned, were concerned about decisions they would have to make about breastfeeding in the future. This puts a mother in a vulnerable position that is not always supported by her surroundings. Women’s agency to handle such a situation is impacted by their own vulnerability and their environment, as well as the support they have around them (Ryan, Team, & Alexander, 2017). Research has also described the experience of early breastfeeding problems as the tension between the lived, embodied experience of struggling to breastfeed and the cultural construction of breastfeeding as “natural” and trouble-free. In such situations, their maternal identities were threatened (Williamson, Leeming, Lyttle, & Johnson, 2012).

Hall Smith, Hausman, and Labbok (2012) found that the current public health promotion of breastfeeding by the World Health Organisation (WHO) relies heavily on physical health messaging and behavioural change at level of the individual mother. Women are told that breastfeeding is the best choice for feeding a child, but too little attention is given to addressing the many social, economic and political factors that, when combined, limit women’s breastfeeding choices. These researchers (Hall Smith et al., 2012) and others (Leeming & Locke, 2017; Regan & Ball, 2013) also point out that too little attention is paid to the issue from the perspective of women. For example, Bartlett found that in recent years, the discourse around breastfeeding has shifted away from women’s own embodied knowledge towards a more biological and medical perspective, which has had a negative impact on a woman’s probability of breastfeeding (Bartlett, 2005). Young (2005) highlighted similar complexities in the western context of female reproduction when arguing that female body experiences, such as pregnancy, birth and breastfeeding, are embodied experiences, e.g., defined by the lived experiences of a human being and not only by the biological processes involved. The embodiment of breastfeeding needs to be considered more thoroughly in order to understand what it means to be a breastfeeding mother. The vulnerability of breastfeeding women is an important part of embodiment and an existential aspect of human life that needs to be further understood (Palmér, Carlsson, Brunt, & Nyström, 2014), and this is why the focus of this study was to further investigate the complexity of breastfeeding for women experiencing difficulties with breastfeeding. This study was part of a larger project focusing on the existential dimensions of breastfeeding from the perspective of women in Sweden. The previously published studies within the project determined that initial breastfeeding difficulties place women as new mothers in exposed and vulnerable situations where they experience loneliness and a feeling of being lost during motherhood (Palmér et al., 2014; Palmér, Carlsson, Mollberg, & Nyström, 2012). Such existential dimensions of breastfeeding difficulties make the relationship with the infant difficult to manage, and the life situation for the new mother can turn into a state of chaos, entailing feelings of inability and doubts about being a mother (Palmér et al., 2012), as well as a sense of insecurity in the breastfeeding relationship (Palmér, Carlsson, Brunt, & Nyström, 2015). Breastfeeding difficulties and the accompanying negative breastfeeding experiences affect a woman’s trust in her ability to breastfeed (Dennis, 2006), and worries over breastfeeding a future child emerge (Larsen & Kronborg, 2013).

Mothers’ breastfeeding decisions and the outcomes of breastfeeding are thus complex issues influenced by diverse circumstances, such as cultural characteristics, availability of care and support, and sense of security and trust in the breastfeeding relationship, among others (Burns, Schmied, Sheehan, & Fenwick, 2010; Palmér et al., 2015; Sheehan, Schmied, & Barclay, 2010). Previous research also highlights breastfeeding and the breastfeeding decision as existential issues (Palmér et al., 2014, 2015; Palmér, Carlsson, Mollberg, & Nyström, 2010; Palmér et al., 2012). In summary, breastfeeding seems to be an important issue for women, and many women experience difficulties. Previous research about experiences of breastfeeding difficulties suggest that this affects women in an existential way, but how these difficulties and their meaning influence thoughts and feelings concerning breastfeeding a future child has not been fully explored and is not well understood. Thus, the aim of the study is to explain the phenomenon of breastfeeding difficulties in order to understand how women’s previous experiences of breastfeeding difficulties relate to their decisions about future breastfeeding.

## Approach and method

A lifeworld hermeneutical approach, developed by Dahlberg, Dahlberg, and Nyström (2008) and Nyström (2017), was used for the study. This lifeworld hermeneutical approach focuses on the world as it is experienced by a human being and the main idea is to explain a phenomenon from this perspective in order to understand it. A phenomenon should, according to this approach, be understood as “something as it is experienced (or lived) by a human being”. The phenomenon in this study is *“how previous breastfeeding difficulties relate to future breastfeeding”* and the aim of the study is to explain the phenomenon of breastfeeding difficulties in order to understand how women’s previous experiences of breastfeeding difficulties relate to their decisions about future breastfeeding.

The epistemological bases for the lifeworld hermeneutical approach used in this study (Dahlberg, Dahlberg, et al., 2008; Nyström, 2017) are grounded in Husserl’s (1950/1981) phenomenological ideas about human consciousness being immersed in natural attitude and intentionality. This means that we as humans take for granted that the world is, as it seems to be for us without questioning it. Human beings are, according to these ideas, understood as thinking and knowing persons experiencing the world “as something”. Such a world is described as the lifeworld. The epistemological ideas through which human beings understand their world are obviously important in a scientific study such as the present one. In this lifeworld hermeneutical study, it is necessary to go beyond the taken-for-granted attitude to adopt a scientific one regarding the phenomenon of “*how previous breastfeeding difficulties relate to future breastfeeding”*. In order to have a scientific attitude in this study, e.g., a phenomenological attitude, Husserl states that it is essential to be aware of this natural attitude and intentionality and to go beyond what has been taken for granted in the natural attitude in order to describe a phenomenon in terms of women’s lived experiences of previous breastfeeding difficulties. Husserl is the origin of the concept of this phenomenological attitude. Accordingly, he pleads the essential importance of having an open attitude in order to really understand something in a new way (Dahlberg, Dahlberg et al., 2008). In addition to the above-mentioned epistemological ideas derived from Husserl, the lifeworld hermeneutical approach used in this study is also based on the epistemological ideas of Hans-George Gadamer (1960/2004) and Paul Ricoeur (1976). Gadamer believes that a self-critical approach towards one’s history and prejudices about a phenomenon, which is, in this study, how previous breastfeeding difficulties relate to future breastfeeding’, is necessary for the openness Husserl advocates, and Ricoeur complements Husserl’s and Gadamer’s ideas about openness by showing how interpretation and explanation are mutually dependent on and inclusive of one another. These ideas, from both Husserl and Gadamer as well as the idea of explanation by Ricoeur, have guided the interpretative work in this study by striving to explain the phenomenon of breastfeeding difficulties, stretching and questioning the natural and the taken-for-granted attitudes about breastfeeding difficulties and their relation to future breastfeeding. Explicitly, the epistemological ideas described above were developed for empirical research as methodological principles that are described as being open and flexible, as well as having a reflective and critical attitude (bridling) towards the phenomenon being studied, in order to grasp the variety of meanings that were used during the entire research process (Dahlberg, Dahlberg et al., 2008; Nyström, 2017). These principles have guided this study’s process of analyzing how women’s experiences of previous breastfeeding difficulties relate to their decision about future breastfeeding.

### Participants

This study was conducted with eight women who expressed that they had experienced severe initial breastfeeding difficulties and, as a result, had sought care and/or support at a maternity ward and/or a breastfeeding clinic. As the aforementioned project and this study focused on breastfeeding difficulties as a phenomenon, the women with lived experiences of this phenomenon were its focus. Breastfeeding difficulties included suckling problems, mastitis, abscesses and psychological conditions, among other problems. The women were recruited for and informed about the study by midwives working at a maternity ward and/or at a breastfeeding clinic.

Seven of the eight women were interviewed on two separate occasions, resulting in 15 interviews. One woman could not be reached for a second interview. The first interview was conducted within 2 months of childbirth, and the second interview was conducted 1.5 to 3 years after the experience of breastfeeding difficulties. At the time of the first interview, seven of these women had one child and one woman had two children. The ages of the women ranged from 20 to 37 years. At the time of second interview, two women had one child, two had one child and were pregnant again, and four had two children, meaning that three of the women had each given birth to a new child since the first interview, and two others would soon. The choice to interview the women on two separate occasions was intended to allow for wider variation in meanings about the phenomenon (Dahlberg, Dahlberg et al., 2008).

### Data collection

This study was conducted through individual lifeworld interviews (Dahlberg, Dahlberg et al., 2008) with eight women who had previous lived experiences of severe breastfeeding difficulties. Interviews were conducted in Swedish by the author of this study. Lifeworld interviews are characterized as being phenomenon-oriented and focused on lived experiences of the phenomenon. The phenomenon under investigation in this study *“how previous breastfeeding difficulties relate to future breastfeeding”*. The interviews started without any predetermined questions except for an open question focusing on the phenomenon: “Please, tell me about your breastfeeding difficulties”. During the interviews, the researcher encouraged the participants to reflect on and describe their personal experiences, thoughts and feelings concerning previous breastfeeding difficulties and especially how they related to their decisions about future breastfeeding. Probing questions were asked to prompt participants to reflect on matters not initially described and to expand their descriptions. Interviews lasted between 45–90 minutes each. Digital recordings of the interviews were transcribed verbatim.

### Data analysis

Analysis was conducted using the lifeworld hermeneutical approach developed by Dahlberg, Dahlberg et al. (2008)and Nyström (2017). The analysis in this study consisted of interpretations in two parts. First, an empirical interpretation based on the interview data related to how women’s previous experiences of breastfeeding difficulties relate to their decisions about future breastfeeding was developed and described in the result section as a main interpretation with six interpreted themes of meanings. Second, the main interpretation was further developed into a philosophical interpretation using Simone de Beauvoir’s (1949/2012) existential philosophy. The two parts of this process of analysis and interpretations are further described below.

### Empirical interpretation

The empirical interpretation was based on empirical material, that is, the transcribed interviews with the participating women. The interpretations were made in order to explain the phenomenon of breastfeeding difficulties in order to understand how women’s previous experiences of breastfeeding difficulties relate to their decisions about future breastfeeding. In explicit terms, the main principle for the analysis was to be attentive to the phenomenon in focus in order to illuminate women’s lived experiences. Throughout the process of analyses, there was an attempt to follow the methodological principles of the lifeworld hermeneutical approach, which is openness and flexibility towards the phenomenon and different ways of interpreting meanings, and includes bridling, having a critical, reflective and problematizing mind towards both the phenomenon and one’s own previous understanding of the phenomenon (Dahlberg, Dahlberg et al., 2008; Nyström, 2017).

The interpretations began when the first whole (the whole set of transcribed text from the interviews) had been read through with an open and reflective mind and read repeatedly to the point where it felt familiar. The process of interpretation was inspired by the hermeneutical spiral (Gadamer, 1960/2004), wherein a movement from the whole (the whole set of interviews about women’s lived experiences) to the parts (interpretations of meanings) and then to the new whole (main interpretation) is performed with an open and reflective mind. In this study, the first stage of interpretation consisted of marking meaning units within the text. Meaning units are parts of the text that carry meaning related to the phenomenon. When all the meaning units and meanings had been identified and structured, they were compared in order to identify patterns of meaning about previous breastfeeding difficulties that belonged together. Similarities within meanings were brought together into interpreted themes of meaning. Six interpreted themes of meaning emerged, each covering one aspect of the phenomenon: *“how previous breastfeeding difficulties relate to future breastfeeding”.*

During the interpretations, an effort was made to maintain openness as well as a critical, reflective and problematizing stance by moving back and forth between the interview data and the six interpreted themes of meaning and comparing them to one another. This was done in order to ensure that the interpretations addressed the significant meanings of the phenomenon and not only the researcher’s own preunderstanding of how previous breastfeeding difficulties relate to future breastfeeding. In an attempt to validate the interpretations, contradictions between interview data and interpretation were investigated during the analysis process. The same process was used to ensure that no information was omitted merely because it did not fit into any of the study’s interpretations. When such inconsistencies were noted, the relevant emerging interpretation was reconsidered, reworded or omitted (Dahlberg, Dahlberg et al., 2008; Nyström, 2017). The interpreted themes of meanings are presented in the result section, highlighted with illustrations from the data, e.g., some statements from the participating women.

Finally, a further comparative analysis among the six interpreted themes gave rise to the empirical part of the main interpretation, which links the six interpreted themes of meanings together. For the main interpretation, the parts (interpreted themes of meanings) were reviewed against the whole (main interpretation) and vice versa. The level of abstraction from the six interpreted themes of meanings to a main interpretation is connected to the principle of moving from parts to the whole and vice versa, striving for consistency in the pattern of interpretations according to the main interpretation. The main interpretation suggests one way to explain and understand how women’s previous experiences of breastfeeding difficulties relate to their decisions about future breastfeeding (Dahlberg, Dahlberg et al., 2008; Nyström, 2017).

### Philosophical interpretation

A philosophical interpretation represents the second part of the analysis within this study and the most abstract part of the interpretation. The choice to perform a philosophical interpretation here was made based on the need to further explain the phenomenon itself in order to understand the hidden and uncovered meanings of how previous breastfeeding difficulties relate to future breastfeeding, as proposed by Ricoeur (1976). In order to develop an interpretation, Gadamer, 1983/1998 suggests that the use of a theory can be a tool for further understanding. For this reason, ideas from the phenomenological and existentialist philosopher Simone de Beauvoir (1949/2012) were used as theoretical support. They were chosen because they offer a fruitful way to deepen the understanding of a given situation for women, in this study this situation being their previous experiences of breastfeeding difficulties.

Even in this part of the interpretation, the methodological principles of openness, flexibility, and reflection, as well as having a critical and problematizing attitude towards the phenomenon and one’s preunderstanding, were essential for ensuring the interpretation offered scientific value (Dahlberg, Dahlberg et al., 2008; Nyström, 2017). The process of the philosophical interpretation was performed in terms of the next step in the hermeneutical spiral (Gadamer, 1960/2004), with a movement back and forth between the empirical part of the main interpretation and its meanings and the philosophical text of Simone de Beauvoir (1949/2012). During the process of interpretation, such a movement was performed in order to open up the horizons of understanding and to achieve a fusion between the meanings in the main interpretation and the philosophical text. De Beauvoir’s philosophical text about what it means to be a woman was used to further illuminate the abstraction of the meanings that were uncovered and were difficult to explain and understand in the empirical part of the main interpretation. Explicitly, during the interpretation there was an encounter between the meanings of the phenomenon in the main interpretation and the meanings of the philosophical texts, from which a deeper understanding of the phenomenon emerged.

### Ethical considerations

Approval and permission to undertake the study were obtained from the Ethics Committee of the Medical Faculty at the University of Gothenburg (recorded as Dnr 373–12). Information about the study was provided verbally and in writing to the participants by the researcher. Participants were informed about the aim of the study, that their participation was voluntary and that withdrawal was possible at any time. All women were assured confidentiality, and the analysis was conducted with the intention of maintaining the integrity of all persons who took part in the study.

## Results

The findings are presented as six interpreted themes of meaning, followed by a main interpretation. The main interpretation consists of two parts, an empirical interpretation and a philosophical interpretation. These two parts should be seen as a whole that suggests how to explain the phenomenon of breastfeeding difficulties in order to understand how women’s previous experiences of breastfeeding difficulties relates to their decisions about future breastfeeding.

## Interpreted themes of meaning

### Fear of reliving an unpleasant experience leads to avoidance of breastfeeding

The experience of previous breastfeeding difficulties results in a fear of repeating that frightening experience, which compels a woman to make a great effort to avoid such a situation with her next child. The potential risk that such an intimidating situation will be repeated creates fear, doubt and worry in that woman’s life. The memory of the actual negative breastfeeding situation activates feelings that in everyday life seem to be repressed, but that are reactivated by thoughts of a new breastfeeding situation. The potential risk of experiencing another intimidating breastfeeding situation can make the idea of breastfeeding a future child so uncomfortable that it is impossible to plan for or implement breastfeeding. The prospect of breastfeeding therefore may feel unreasonable and overly stressful throughout the pregnancy. A pre-emptive decision not to breastfeed a future child may allow a woman to find peace in a planned or present pregnancy. As one woman noted:

I think about breastfeeding several times a week. I am totally scared of life. I would go all way through pregnancy and be agitated over breastfeeding. Therefore, I choose not to breastfeed again. I do not want to experience that again. It is the worst thing that has happened in my life.

Previous experiences of bad and objectifying care that can also contribute to fears around breastfeeding and create worries that such experiences will be repeated in future breastfeeding. The lack of confidence or trust in healthcare professionals causes a woman to doubt her ability to breastfeed or to avoid future breastfeeding entirely. One woman expressed this fear: *I cannot breastfeed again. I do not dare to trust healthcare professionals anymore because they exposed me to terribly bad care where I was left to care to myself and treated as if I was not worth caring for.*

### Pregnancy and breastfeeding reawakens one’s repressed memories

A new pregnancy, the initiation of new breastfeeding or the sight of a pregnant or breastfeeding woman reawakens the memory of previous breastfeeding experiences. The great effort made to forget negative breastfeeding experiences may result in unprocessed experiences that are preserved in the body as memories, making it difficult to forget or override the associated negative feelings. The memory of previous breastfeeding experiences is retained and remains, despite efforts to repress it. Repression is interpreted as a defence mechanism, as an attempt to forget the previous experiences of breastfeeding difficulties. That negative memory, however, can be brought back when something happens to activate it, such as a new pregnancy or seeing a pregnant or breastfeeding woman. The memory can be so powerful that, despite the fact that several years have passed since breastfeeding occurred, it affects present life. One woman described such a memory: *I feel the horrible feeling again. It came back when I started breastfeeding. It seems that the body remembers.*

If, during previously experienced breastfeeding difficulties, the experience turned into a positive one, the memory of the first, most difficult time may recede, and the positive memory of the breastfeeding experience pre-empts the negative one. Such an experience may make future breastfeeding possible. One woman stated: *At the end, after huge painful struggling, it was easy breastfeeding my previous child. So that’s what I remember best and take with me.*

### Desiring a close relationship with one’s child can influence the breastfeeding decision either way

There is a desire to breastfeed again in order to create closeness with the child through a very special way of being together that is perceived as being unique to harmonious breastfeeding. Such closeness is perceived as a way to promote well-being, making life enjoyable and secure for the infant as well as for oneself as a mother. These thoughts re-emerge when considering whether or not to breastfeed another child. One woman expressed this: *The few times I really managed to breastfeed him, it was a cosy feeling that makes me want to try again.*

Along with the desire to breastfeed, there is the intimidating feeling of how past breastfeeding difficulties caused a distance and loss of closeness with the child. The risk of repeating this felling makes it difficult to consider future breastfeed. When the process of creating that closeness is most strongly associated with problematic feelings in the woman, it can make a future breastfeeding situation seem unthinkable. One woman expressed this: *The first months of the child’s life just disappeared last time. I want a harmonious relationship with the next child that comes, but when breastfeeding is not working, it is hard and demanding for the relationship with the child.*

### Learning from one’s experiences allows taking command

Experiences from previous breastfeeding difficulties may provide the preparedness necessary to take command of a future breastfeeding situation. Preparedness is interpreted as having confidence to actively deal with both the practical and the emotional aspects of breastfeeding in a future situation in order to have a better experience than the previous one. Previous negative breastfeeding experiences can be ameliorated by the creation of new memories that are characterized by a feeling of independence and capability of managing breastfeeding on one’s own. The ability to compare experiences may become a positive force when taking command and daring to breastfeed again. One woman expressed this: *She (the first child) was even harder than he was. I was more mentally prepared that it might be trouble… I knew what was required from me and could handle breastfeeding positions with ease.*

Experiences of previous care during breastfeeding can provide preparedness for managing different ways of being cared for. That knowledge makes it possible to take command in order to get the care necessary for one’s individual needs and concerns. Taking command also means daring to make demands upon the care system, not just accept poor or non-existent care.

### Telling one’s breastfeeding story eases suffering

Telling one’s breastfeeding story and reflecting on previous breastfeeding experience allows a release of feelings that eases suffering, which appears to deepen the understanding of what happened. If these experiences are not processed, negative feeling may become overwhelming. Thus, the process of storytelling is experienced as beneficial for well-being, independent of the decision about future breastfeeding, and it gives a sense of security, sometimes encouraging a woman to breastfeed in the future and to reconcile memories of a period of suffering in her life. The experience can be as one woman described it: *I have been telling my breastfeeding experiences and reflecting with my midwife over my experiences. It makes me understand what happened, and I feel more secure.*

Telling one’s breastfeeding story might provide an opportunity to plan for future breastfeeding by writing down the breastfeeding story, as well as by formulating wishes based on previous experience. Being able to write down one’s breastfeeding story and thus express one’s wishes for the future can be a powerful way to protect oneself from again being subjected to poor care in the form of intrusive hands-on breastfeeding instruction, objectifying care or other forms of inhumane care. The woman further noted: *I will write in a letter that no one should come in and do this unpleasant hard way of caring. I will get guidance… not intrusive hands-on breastfeeding help.*

### Reformulating breastfeeding makes it possible to proceed with life

The meaning of breastfeeding can be reformulated in light of how previous breastfeeding was experienced, how the process was understood and how breastfeeding ended. By toning down the emphasis on the importance of breastfeeding, the level of intimidation around the idea of being a breastfeeding mother may be reduced, allowing the possibility of breastfeeding to be considered. With distance from previous breastfeeding experiences, the importance of breastfeeding in the mind of a woman can be reformulated, with the previous difficult experience becoming a conscious motive for not breastfeeding. The revision might also act as a protection against other people’s views and hard words, as one woman noted:

Now, afterwards, I have a distance. I would not be as sad as I was previously. But it is etched as a very hard memory of being a bad mother. Now I know that it can go wrong… I have gone beyond this because now I know I am not that bad or insufficient if I am not able to breastfeed.

### The main interpretation

The interpretation suggests that women with previous experiences of breastfeeding difficulties find themselves in vulnerable situations, as both women and mothers, in which the lingering memories from previous breastfeeding create an existential breastfeeding trauma in an individual woman’s life that follows her through motherhood in the form of an embodied memory for several years after the cessation of the actual breastfeeding situation. The interpretation of the existential breastfeeding trauma suggests two intertwined pathways for future breastfeeding––fear and longing––that affect how women think and feel about breastfeeding in the future. Previous experiences, therefore, have both the potential to enable and to threaten by giving the power to try breastfeeding again and by draining it too. In the worst scenarios, doubt, uncertainty and insecurity develop into a fear of being forced into or of forcing oneself to once again endure and suffer the demands of breastfeeding. Such a development seems to be further strengthened by the experiences of objectifying care, wherein being abandoned, treated like an object, mistreated or being left alone are overwhelming and hard experiences to deal with. This overwhelming memory gives rise to doubt about whether it is worth it to try to breastfeed again. A previous difficult breastfeeding experience and a negative experience of care may therefore hinder a woman’s fulfilment of her inner wishes for a harmonious breastfeeding relationship with her future child. Previous experiences of breastfeeding difficulties may not always induce overwhelming feelings of fear towards breastfeeding; they can also induce a longing to breastfeed a future child. Such longing retains a sense of security and trust in one’s ability to breastfeed and mother the child despite previous difficulties. The possibility of telling one’s breastfeeding story and reflecting on past experiences has the potential to ease suffering, creating a sense of security and trust in oneself and the infant, as well as providing an understanding of what happened and why this is emotional. The two paths of fear and longing are intertwined with each other in ambiguous ways in an individual woman’s life. If the fear of breastfeeding is stronger, the idea of future breastfeeding may seem unthinkable, even though a longing for breastfeeding exists; future breastfeeding remains a likely possibility, despite the lingering fears, but it depends on how fear and longing are balanced with one another in a woman’s life. This ambiguity is not interpreted as having a causal explanation but rather an existential one.

### Philosophical interpretation

In this section, the philosophical interpretation is presented in order to increase for understanding why previous breastfeeding difficulties have an existential dimension, with the potential to create an existential trauma of intertwined with fear and longing. In this study, this is further interpreted using the philosophical thoughts of the existential philosopher Simone de Beauvoir (1949/2012), related to the facticity of being a woman and how women are situated in given contexts, which affects their potential for freedom and choice in life. A situation, in terms of existential philosophy, contains things that are given, things the woman cannot change, such as place of birth, traditions and historical aspects of life, but it also involves a freedom to, in every moment, choose what to do with such givens. De Beauvoir states that each woman is always free to choose the direction in life. De Beauvoir denies that women makes choices based solely on their biological nature. A woman only acquires a nature insofar as she commits herself to certain ways of acting through the choices she makes. Nor does she make choices based on her values. Rather, by making choices, she commit herself to particular values. Thus, a woman chooses to breastfeed, and in so, doing she commits herself to the idea that breastfeeding is valuable. According to the situatedness of a breastfeeding woman, this choice reflects biological, cultural, economic and social aspects, but it is also political and existential. Within this idea, de Beauvoir regards the woman as a lived body, immersed in her cultural context, and not just as a determined biological body. For de Beauvoir, the lived body is not a set of biological facts but a set of meaningful facts whose meaning is derived from choices made over time. If a woman chooses to breastfeed, the meanings of this choice become embodied in her body—perhaps breastfeeding signifies to her that she is a good mother based on prevailing norms. In relation to previous breastfeeding difficulties and the meaning of such experiences for future breastfeeding, the situatedness can be interpreted as being in a lifeworld situation wherein the previous experiences are intertwined with each other in ambiguous ways in an individual woman’s life. In the intertwining, the experiences and feelings are interwoven in a complex pattern wherein the freedom to choose to breastfeed again is explicitly based on the givens of the previously experienced breastfeeding, but it is also based on the prevailing norms in western culture about how to be a woman and a mother. In such a given situation, one’s own inner wishes to become a breastfeeding mother may be overwhelmed by the feelings of existential trauma and fear. These wishes may also be disrupted by feelings of vulnerability in the breastfeeding relationship and the desire to live up to existing norms and cultural values about how to be a mother, in which every woman that becomes a mother is immediately immersed. The decision to breastfeed can also depend on the nature of the care that a woman receives. In the best case, she has a caring relationship, wherein caring care has her breastfeeding story as a focus. In the worst case, no caring relationship exists, which would be described as objectifying care in which a woman is abandoned, treated like an object, mistreated or left alone. In this exposed situation it is hard to be true to one’s own inner values about breastfeeding.

The idea of situatedness implies an ambiguity to life as it sets limits on a woman’s ability to make individual breastfeeding choices in everyday life. In western culture, where a determined view of women as biologically driven objects exists, the limitations on women’s freedom can be explicitly understood to be situated in a care context with objectifying and inadequate care, where breastfeeding is seen as being the biological responsibility of an individual woman due to her biological nature, as well as being an easy and natural event in life. In a care context in which a woman is situated mostly as a reproductive and biological object and breastfeeding is promoted as a norm for public health, as well as a medical issue, but is not understood as an existential experience, there is a risk that a woman will internalize the cultural view that women are unfree beings, trapped in their bodies by their milk-producing breasts, unable to choose freely because their biology compels them to act in certain ways. De Beauvoir means that women have accepted and internalized a view that they are defined by their bodies and by fixed characteristics such as the idea that a good mother (caring, motherly, womanly) should breastfeed and not doing so means she is a bad mother (selfish, ego, lazy). When women do not live up to the ideal of being a breastfeeding mother, they might be overwhelmed by the prevailing norms, which are the basis for the development of the existential breastfeeding trauma in this interpretation. The interpretation suggests that a woman perceives herself trapped in her own body, which she feels is biologically unworthy in relation to breastfeeding mothers who do not experience problems. De Beauvoir explains that women tend to accept this objectified view of themselves because that view is pervasive in western culture. Women appear to be oppressed in situations wherein oppression can be defined as being deceived or tempted into not exercising one’s freedom, being defined as a breastfeeding woman by nature not by choice. The interpretation therefore suggests that an individual woman is not solely responsible for her breastfeeding decision. Instead, the responsibility lies with the situation of the care context and the lack of adequate care available to women who want to breastfeed and are experiencing difficulties. The current ideology about care for breastfeeding women carries a risk that breastfeeding could be seen as the biological responsibility of an individual woman, which may force her to accept complete responsibility for previous breastfeeding difficulties, thus inducing her with a sense of downgrading herself as a bad mother and resulting in the development of a fear of breastfeeding.

The philosophical interpretation suggests that the existential breastfeeding trauma can be understood in relation to the woman’s given situation, e.g., western culture, and the meaning and value that an individual woman gives to breastfeeding and her previous breastfeeding experiences. In accordance with de Beauvoir’s ideas, the philosophical interpretations of this study suggest that a woman can feel trapped in her body when it is situated in a care context in which only the biological properties of a woman determine the care she receives. In such a care context, her freedom and choices in respect to breastfeeding may be limited. The solution, according to the existential philosophy of de Beauvoir, might be for women (and other people in the western culture such as carers) to embrace the ambiguous condition that all human beings are immersed in: the freedom to create one’s life and values and freedom of choices in life, as well the boundaries set by culture. In terms of how to care for women with previous experiences of breastfeeding difficulties, this interpretation suggests that the ambiguity of being free to choose breastfeeding and being forced by the prevailing norms, as well as the ambiguity of fear and longing, must be taken into consideration in order to develop caring care practices. Furthermore, breastfeeding must be embraced as a conscious, meaningful act, not merely a biological duty that a woman performs because it is part of her biological nature as a woman.

## Discussion

This study contributes to the body of knowledge about how women’s previous experiences of breastfeeding difficulties relate to their decisions about future breastfeeding, emphasizing that such experiences entail an existential breastfeeding trauma with two intertwined pathways for future breastfeeding: fear of breastfeeding and longing for breastfeeding. Thus, an understanding of the existential dimensions of previous breastfeeding experiences, as well as the knowledge of the consequences of breastfeeding women’s situatedness in a care context immersed in the objectifying view of women as by nature driven by their biological bodies instead of free choices that is pervasive in Western culture, are important for the development of caring practices.

Researchers from different disciplines describe the context of breastfeeding from a western perspective (e.g., European and North American perspectives). Most often, breastfeeding has been considered within biological and technical contexts, and a deterministic view has predominated, describing breastfeeding as an easy, natural function—something that all women can do if they want to, something that does not require special care as it is a natural biological phenomenon. Such ideas are without considering the consequences of women’s breastfeeding experiences and decisions (Bartlett, 2005; Hall Smith et al., 2012; Larsen & Kronborg, 2013; Leeming & Locke, 2017; Palmér et al., 2015; Regan & Ball, 2013; Williamson et al., 2012; Young, 2005). In the context of Sweden, where this study was performed, as well as the contexts of Denmark (Larsen & Kronborg, 2013), England (Williamson et al., 2012) and the US (Hall Smith et al., 2012), breastfeeding is considered to be both a biological and cultural norm and the gold standard for infant feeding (WHO, 2013). Promoting breastfeeding as the gold standard is, in many ways, very important for protecting women’s right to breastfeed if they want breastfeed (Palmér et al., 2015). But this study, as well as others (Hall Smith et al., 2012; Johnson, Williamson, Lyttle, & Leeming, 2009; Larsen, Hall, & Aagaard, 2008; Larsen & Kronborg, 2013; Palmér et al., 2015; Williamson et al., 2012), found that such policy-level recommendations are not enough to provide care for individual women who have experienced breastfeeding difficulties. The results of this study indicate a risk inherent in a normal component of the foundation of care, the view that breastfeeding is a simple and natural biological process. In such situations of care, there is a risk that women are left alone to handle their breastfeeding difficulties, with bad, incompetent and objectifying care, and without the adequate caring needed to make breastfeeding work. The difficulty of the experience can affect women’s lives in profound ways through existential trauma and the risk of developing a fear of breastfeeding.

This study also suggests that there is sometimes a mistrust of professional healthcare providers due to past negative experiences. Such issues are of value to reflect upon, as care itself seems to be a risk factor for not breastfeeding a future child. In an ethnographic study about women’s breastfeeding difficulties, Hauck, Langton, and Coyle (2002) describe two paths of determination that women experience, expressed as “staying on the path” and “coming off the path”, and the care received is an important factor influencing the choice of path. This description has similarities to this study’s description of two intertwined pathways for future breastfeeding, which makes this worth considering for care development. This study determined the importance of viewing issues related to women’s reproduction, including breastfeeding, as concepts larger than simple biologically function. Reproductive issues, such as breastfeeding and being a new mother, are existential in nature (Palmér et al., 2014, 2015, 2010, 2012; Prinds, Hvidt, Mogensen, & Buus, 2014). This nature must be taken into consideration when caring for women who have experienced difficulty breastfeeding in the past; the discussion of care development for women during the transition to motherhood and during the breastfeeding period should, involve the existential nature of reproductive issues. According to this study, it is of obvious importance that health care professional have a caring approach which consider and value the existential aspects of women’s breastfeeding experiences. Such a model of care for breastfeeding women does not currently exist in the healthcare system in Sweden. Caring from a caring science perspective based on a lifeworld approach as the foundation for care (Dahlberg, 2011; Dahlberg & Segesten, 2010) has the potential to develop a model of care sensitive to existential dimensions. Such an approach is grounded in Husserl’s (1977/1929) lifeworld theory and theory of intentionality and Merleau-Ponty, 1945/2011 further development of the lifeworld theory, which clarifies human beings’ existence in the world as “lived bodies”. Merleau-Ponty, 1945/2011sees the human being as an integrated whole, a lived body with no possible division between body and soul. This is in line with de Beauvoir’s (1949/2012) philosophical ideas of the lived body used in this study to develop the understanding of women’s previous experiences of breastfeeding difficulties. The concept of the lived body according to de Beauvoir (1949/2012) captures the fact that women always experience their female bodies as meaningful. One’s body never presents itself as a set of bare biological facts. de Beauvoir (1949/2012) develops the idea of the lived body when she suggests that women always been viewed as the “other” of men. In fact, women have let men define them as beings with an essence—women’s nature—rather than as subjects who exists, who are responsible for choosing the course of their own lives. Thus, women exist in a western culture that merely defines women according to their biological bodies, and both men and women internalize this thinking. A health care professional’s ability to provide care for a woman may be hindered by the internalized view of women as biological driven objects. Ekebergh (2009) suggests that in order to be aware of one’s own taken-for-granted attitudes as a carer, one must employ a reflective attitude towards oneself, and, in relation to this study, towards breastfeeding, allowing the carer to approach a woman’s lifeworld as well as their own. In relation to this study, this is of obvious importance since the encouragement of reflection upon women’s and carers breastfeeding experiences needs to be done with respect and without judgment in order to be caring and ease suffering. According to Galvin and Todres (2009), it is important to understand oneself as a carer in order to have the ability to understand another human being. They argue that carers own embodied memories awaken when caring for a patient e.g., a breastfeeding woman, which may make the carer more or less caring. At the same time, there must be an understanding that one can never fully understand another human being. Instead, there must be a willingness and openness towards the other person’s experiences (Todres, Galvin, & Dahlberg, 2014). Carers that provide care for breastfeeding women therefore needs to reflect their own attitude and meanings about breastfeeding in order to be open and sensitive towards the women’s experiences.

In the understanding of woman as a lived body (de Beauvoir, 1949/2012; Merleau-Ponty, 1945/2011), it is impossible to separate a woman from her breastfeeding, i.e., we cannot separate a woman from her experiences of and the meanings and values she gives to breastfeeding. In order to see and understand a woman, health care professionals need to embrace a caring attitude signified by curiosity, openness and willingness to encounter the woman’s lifeworld in order to let the woman’s own story provide the foundation for her care (Dahlberg & Ekman, 2017). In such an approach, caring is driven by the patient’s perspective, which means that the woman’s perspective, i.e., the woman’s lifeworld, is the foundation for caring (Dahlberg, 2011; Dahlberg & Segesten, 2010; Galvin & Todres, 2013). The focus for caring within such an approach is on the development of professional care from a woman perspective. That will enable woman to feel well and carry out her life projects, e.g., give her own value to breastfeeding and breastfeed according to this value, and will strengthen health processes based on the individual woman’s needs and desires (Dahlberg, 2011; Dahlberg & Segesten, 2010; Galvin & Todres, 2013).

This study highlights the importance of a lifeworld-led caring approach (Dahlberg & Segesten, 2010; Dahlberg, Todres, & Galvin, 2008; Galvin & Todres, 2013; Todres, Galvin, & Dahlberg, 2007) to understanding breastfeeding as an existential issue and reflecting on a woman’s breastfeeding story as part of caring for a pregnant and/or breastfeeding mother. Such approach may enhance a woman’s well-being and ease her suffering despite choosing to breastfeed a future child or not. The study also indicates that the breastfeeding story based on a lifeworld approach presents the possibility of giving voice to the existential dimensions of breastfeeding in a way that eases suffering from previous experiences. Otherwise, the cultural attitudes around breastfeeding as only a biological and technological event will persist (Regan & Ball, 2013), and the existential dimensions of breastfeeding will not be heard. The political and feminist philosopher, Iris Marion Young, in her essay entitled “A Breasted Experience”, argues that women’s breasts are seen as disembodied and sexualized objects (Young, 2005). In this context, breastfeeding as an existential phenomenon is overridden, and women are viewed as biologically driven objects rather than existential ones. Smith, Hausman and Labbok (2012), as well as Van Esterik (1994), suggest that breastfeeding is an important feminist issue, an understanding that is supported by the results of this study. Previous research and this study together highlight the need for the cultural context and the conditions of the care provided, not only an individual woman’s capacity to breastfeed, to be the primary focus in developing the necessary conditions for breastfeeding to be a positive experience (Hall Smith et al., 2012; Larsen & Kronborg, 2013; Williamson et al., 2012). Otherwise, as indicated by Galvin and Todres (2013), that traumatizing event can be a source of overwhelming temporal suffering that significantly reduces a person’s well-being.

### Methodological reflections

Finally, to evaluate the methodological basis for a lifeworld hermeneutical study, it is important to understand that lifeworld hermeneutical research is inherently contextual; therefore, the conclusions of this study should be considered in light of the context (Dahlberg, Dahlberg et al., 2008), women in Sweden with previous experiences of breastfeeding difficulties. In order to have variations in the phenomenon (Dahlberg, Dahlberg et al., 2008), it became necessary to carry out interviews during the actual period of breastfeeding difficulties but also some years later. During both interviews, the participating women provided the researcher with rich descriptions of the experiences around breastfeeding. From a positivistic view, the risk of a negative recall factor could still be a limitation, yet it was clear from the interview data that breastfeeding difficulties affect women in such ways as to make them acutely memorable, but the women had no problems talking about their previous experiences. The important notion is that lifeworld studies are phenomenon-oriented, focusing on meanings of the lived experiences: “the truth” is in these meanings regardless of the amount of time since the experiences (Dahlberg, Dahlberg et al., 2008).

Moreover, the researcher’s efforts to maintain the methodological principle of openness and flexibility towards the phenomenon included gaining awareness of the possible influence from what Gadamer (1960/2004) calls preconceptions and prejudices, and it is important to understand the impact of those preconceptions and minimize the potential for misleading, prejudiced interpretations as a result of them. According to Gadamer, it is not possible for a researcher to present his/her preunderstanding since the preunderstanding becomes apparent in relation to data and the interpretations the researcher makes. Gadamer (1960/2004) explains that the researcher must provoke one’s preunderstanding and put it “at risk” during the research process. The researcher in this study did this by constantly questioning the interpretations, by asking questions of the data and the interpretations made to put the researcher into an open frame of mind. Therefore, there has been an attempt to question and thus maintain an open mind throughout the research process. This was accomplished through individual reflection and reflections by other researchers, as well as by practicing the methodological principles with close attention to the interview data and the meanings of the phenomenon described (Dahlberg, Dahlberg et al., 2008).

The choice to perform a philosophical interpretation stems from the possibility of further developing the understanding of the phenomenon by uncovering hidden meanings in the empirical data (Ricoeur, 1976). In this study to further uncover hidden meanings, explain and develop the existential dimensions described in the empirical interpretation in order to increase their value for caring practice. This was done carefully with immense respect for both the empirical results and the philosophical texts, and the philosophical interpretation presented in this study represent one possible way to further explain and understand the phenomenon. The process of the philosophical interpretations started when openness to the phenomenon was emphasized in the first stage, as suggested by Gadamer (1960/2004).

## Conclusion and implications for caring practice

To conclude, this study suggests that previous breastfeeding difficulties may constitute a traumatic existential experience that a woman might carry with her in motherhood as a negative breastfeeding experience, etched into her life as an embodied memory. Such experiences might have profound impacts on women’s lives several years after the actual breastfeeding situation. As such, it is an important issue for caring practice to consider. Implications for caring practice include the need for carers to embrace the ambiguity of breastfeeding as a meaningful act affected by both fear and longing and the need to reflect on how they give care, which meanings they give to breastfeeding and in what ways these meanings are influenced by the western view of women as biologically driven objects. Such a caring approach should be based on a lifeworld-oriented caring science perspective, which has the potential to contribute to care that considers the existential dimensions of the breastfeeding trauma. Women with previous experiences of breastfeeding difficulties should therefore be offered caring based on a lifeworld-oriented caring science perspective with the breastfeeding story as the ethical compass for caring. Such a foundation provides an opportunity for women to reflect on feelings, past experiences and wishes for the future in order to ease the suffering of the existential breastfeeding trauma, prevent a fear of breastfeeding and strengthen their own well-being.
